# Development of an immunochromatographic lateral flow assay for the rapid detection of all known orthoebolavirus glycoproteins

**DOI:** 10.1038/s44298-026-00209-1

**Published:** 2026-06-24

**Authors:** Helene Schulz, Guodong Liu, Jordan Wight, Karla Emeterio, Wenguang Cao, Mable Chan, Ming Yang, Logan Banadyga

**Affiliations:** 1 Special Pathogens Program, National Microbiology Laboratory, Public Health Agency of Canada, Winnipeg, MB Canada; 2 National Centre for Foreign Animal Disease, Canadian Food Inspection Agency, Winnipeg, MB Canada; 3 Department of Medical Microbiology and Infectious Diseases, University of Manitoba, Winnipeg, MB Canada

**Keywords:** Biological techniques, Diseases, Microbiology

## Abstract

Orthoebolaviruses, like Ebola, Sudan, and Bundibugyo viruses, are responsible for causing sporadic and unpredictable outbreaks of severe disease throughout Sub-Saharan Africa. Accurate and reliable diagnostic tests are key to identifying cases of disease and restricting its spread, yet options are mostly limited to molecular assays, such as reverse transcriptase quantitative PCR, that require time, training, and complex equipment to run. To complement existing diagnostic modalities, and to address some of their shortcomings, we developed a prototype immunochromatographic lateral flow rapid diagnostic test (ILF-RDT) that detects the glycoproteins (GPs) from all known orthoebolaviruses, including Bundibugyo virus, which is currently causing a large outbreak in the Democratic Republic of the Congo and Uganda. This test depends on a pair of novel, broadly cross-reactive monoclonal antibodies capable of binding the viral GP—including the secreted GP—in a non-competitive manner. Here, we show that this ILF-RDT detects both recombinant GP and authentic virus in a variety of substrates, including samples spiked with human blood and blood samples taken from experimentally infected animals. Indeed, the ILF-RDT was able to detect Ebola virus in nonhuman primate samples at 4 days post-infection, before the animals began exhibiting signs of severe disease. Given the uniquely broad cross-reactivity of this diagnostic assay, its ability to detect low levels of virus early during infection, and its suitability for use at the point-of-care in resource-limited settings, this ILF-RDT could have a significant impact on the management and mitigation of future orthoebolavirus outbreaks.

## Introduction

Orthoebolaviruses comprise a genus of viruses that belong to the family *Filoviridae*^1^. Ebola virus (EBOV), Sudan virus (SUDV), and Bundibugyo virus (BDBV) all cause severe disease in humans, with EBOV, in particular, having caused numerous outbreaks across Africa since its discovery in 1976. As a result, these viruses pose a significant threat to global health. Taï Forest virus (TAFV), another orthoebolavirus, has caused only a single documented non-fatal case of human disease, while Reston virus (RESTV) appears to be apathogenic in humans^2^. The pathogenic potential of the newly discovered Bombali virus (BOMV) is still unclear; however, preliminary evaluation in humanized mice suggests that it may also be apathogenic in humans^3^. The emergence of orthoebolaviruses in human populations is thought to be the result of zoonotic spillover from a currently unknown animal reservoir, although bats are strongly suspected^4^. Given the unpredictable nature of these outbreaks, along with the high pathogenicity of these viruses and their ability to spread rapidly from human to human, the World Health Organization (WHO) has identified orthoebolaviruses as Priority Pathogens requiring urgent research^5^.

Disease caused by orthoebolaviruses is referred to as Ebola disease (EBOD) and is characterized by symptoms typical of viral hemorrhagic fevers, including fever, fatigue, myalgia, abdominal pain accompanied with vomiting and diarrhea, and, occasionally, bleeding^1,4^. Frequently, disease progresses to multiple organ failure and death, with case fatality rates ranging between ~30 and 90%, depending on the virus and the outbreak^1,4^. To date, the most devastating outbreak of EBOD occurred in 2013–2016, primarily across the Western African countries of Sierra Leone, Liberia, and Guinea, when EBOV infected nearly 30,000 people and killed over 11,000^6^. A subsequent outbreak of EBOV in the Democratic Republic of the Congo (DRC) from 2018 to 2020 resulted in over 3000 cases and over 2000 deaths^7^. Since then, both EBOV and SUDV have re-emerged multiple times, with SUDV having caused a small outbreak in Uganda in early 2025 and EBOV having caused an outbreak in the DRC later in the same year^8,9^. At present, a large outbreak of BDBV is currently ongoing in the DRC and Uganda, with at least 900 suspected cases and 220 deaths reported as of May 25, 2026^10^.

Accurate, efficient, and rapid diagnostics are essential tools for the identification of infected individuals and the management of outbreaks^11–13^. Molecular assays based on reverse transcription quantitative PCR (RT-qPCR) are the gold-standard and most widely used diagnostic test for orthoebolaviruses^14^. These tests are highly sensitive and specific in the detection of orthoebolavirus RNA; however, they are resource intensive, require highly trained personnel and specialized equipment/reagents, and can take several hours to return results. Additional diagnostic methods, such as loop-mediated isothermal amplification (LAMP) and cartridge-based molecular biology devices, such as the Cepheid^®^ Xpert^®^ Ebola Assay and BioFire^®^ FilmArray^®^ BioThreat-E test, have been developed to mitigate some of these requirements and are easier to perform with limited training^15–18^. However, cartridge-based tests are expensive and take about 60 to 90 min to provide results^19,20^. In addition, all molecular assays can be challenging to use in resource-limited settings as they require a consistent power source. As such, there is a need for diagnostic assays that are more amenable to point-of-care application and that can provide rapid and accurate results.

Immunochromatographic lateral flow (ILF) assays, also known as strip tests, are a type of rapid diagnostic test (RDT) that can provide quick and accurate diagnoses at the point-of-care with little training and a limited requirement for additional resources, such as cold chain or electricity^21^. ILF assays have been increasingly used for detection and diagnosis of a variety of pathogens, including SARS-CoV-2, Influenza A and B, respiratory syncytial virus, and EBOV^22^. These assays typically rely on highly specific and sensitive monoclonal antibodies (mAbs) to bind an antigen in a given sample and produce a visual result within 15 to 30 min. Often enclosed within a plastic cassette, the test strip consists of multiple different components (Fig. S1). A liquid sample (with or without buffer) is directly applied to the sample pad at one end of the strip and is drawn across a nitrocellulose membrane via capillary action driven by the absorption pad at the opposite end of the strip. Prior to entering the nitrocellulose membrane, the sample moves through the conjugate release pad, which holds the detection mAb conjugated to a signal-producing moiety, such as gold nanoparticles. The detection mAb binds to its cognate antigen, if present in the sample, and this immune complex then enters the nitrocellulose membrane. At the proximal end of the membrane is the “test line”, containing immobilized capture mAb, which binds to the antigen in a manner that does not compete with the detection mAb. Binding of the antigen-detection mAb immune complex results in the concentration of the signal-producing moiety at the test line, which can then be visualized by eye. Excess detection mAb continues to flow along the nitrocellulose membrane until it encounters a third mAb, immobilized at the control line, that is specific for immunoglobulin. Formation of a visible band at the control line confirms the correct flow of the assay. A positive diagnostic result is indicated by a visible band at both the test and control lines, while a negative result is indicated by a band only at the control line. Although ILF assays often have a lower sensitivity than molecular-based tests, their ability to provide rapid but accurate diagnostic results—particularly in symptomatic patients—permits quick case identification and swift implementation of public health measures.

Using previously generated murine mAbs specific for orthoebolavirus glycoproteins (GPs)^23^, we developed a sensitive and broadly reactive ILF-based RDT that is capable of detecting all known orthoebolavirus GPs. This assay was tested using both recombinant GPs and authentic viruses in a variety of media, including human whole blood and serum. The assay was also able to detect virus in blood samples collected from experimentally inoculated lab animals, including nonhuman primates (NHPs). To our knowledge, this ILF assay—although still just a prototype—is the first to be able to detect all known orthoebolaviruses (i.e., EBOV, SUDV, BDBV, TAFV, RESTV, and a recombinant EBOV expressing BOMV GP), making it potentially useful for managing future outbreaks.

## Results

### Identification of suitable antibodies for an orthoebolavirus-specific RDT

**Fig. 1:**
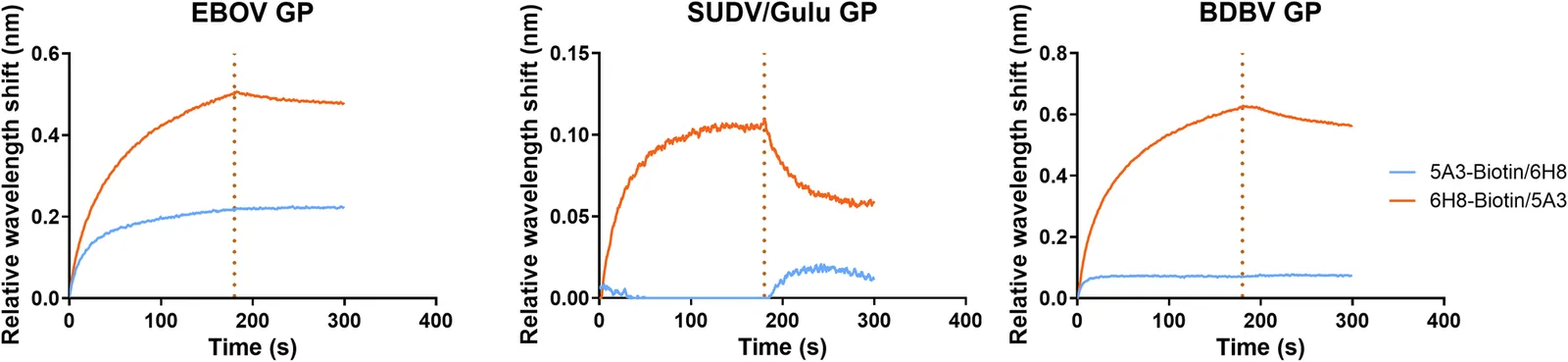
Bio-layer interferometry (BLI) confirmed the selection of 5A3 as capture mAb and 6H8 as detection mAb for the pan-orthoebolavirus ILF-RDT. A Sartorius Octet instrument was used to quantify the sequential binding of antibodies to recombinant EBOV, SUDV, and BDBV glycoproteins (GPs). Streptavidin sensors loaded with biotinylated mAbs (representing gold-conjugated detection mAbs) were exposed to GP, followed by exposure to a second, unconjugated mAb (representing the capture mAb). The association and dissociation of the second mAb was quantified. Values in this figure represent the binding signal of the second mAb, with higher values indicating that binding of the first antibody did not restrict binding of the second. The x-axis displays time of the association and dissociation steps in seconds (s), separated by the dotted line; the y-axis displays the relative wavelength shift in nm caused by the binding of the unconjugated mAb.

We previously generated and characterized multiple murine mAbs that showed broad cross-reactivity against several different orthoebolaviruses^23^. Of these mAbs, six were selected for potential incorporation into an ILF-based RDT. To identify suitable detection and capture mAb pairs, we evaluated all 36 potential combinations of the six mAbs via a competitive biolayer interferometry (BLI) assay against recombinant GPs from EBOV, SUDV, and BDBV (Fig. 1 and Fig. S2). All three GPs bound to biotin-tagged mAb 6H8 (representing the detection antibody), which did not inhibit subsequent binding of mAb 5A3 (representing the capture antibody). Notably, the reciprocal arrangement—in which 5A3 was biotinylated and represented the detection antibody while 6H8 represented the capture antibody—did not permit sequential binding of both antibodies to all three GPs (Fig. 1). No other mAb pair showed binding efficiencies as high as biotinylated 6H8 and 5A3 across all three GPs (Fig. S2). Thus, 6H8 was chosen as the detection antibody and 5A3 was chosen as the capture antibody for our ILF-RDT assay.

### The ILF-RDT detects EBOV, SUDV, and BDBV GPs

**Fig. 2:**
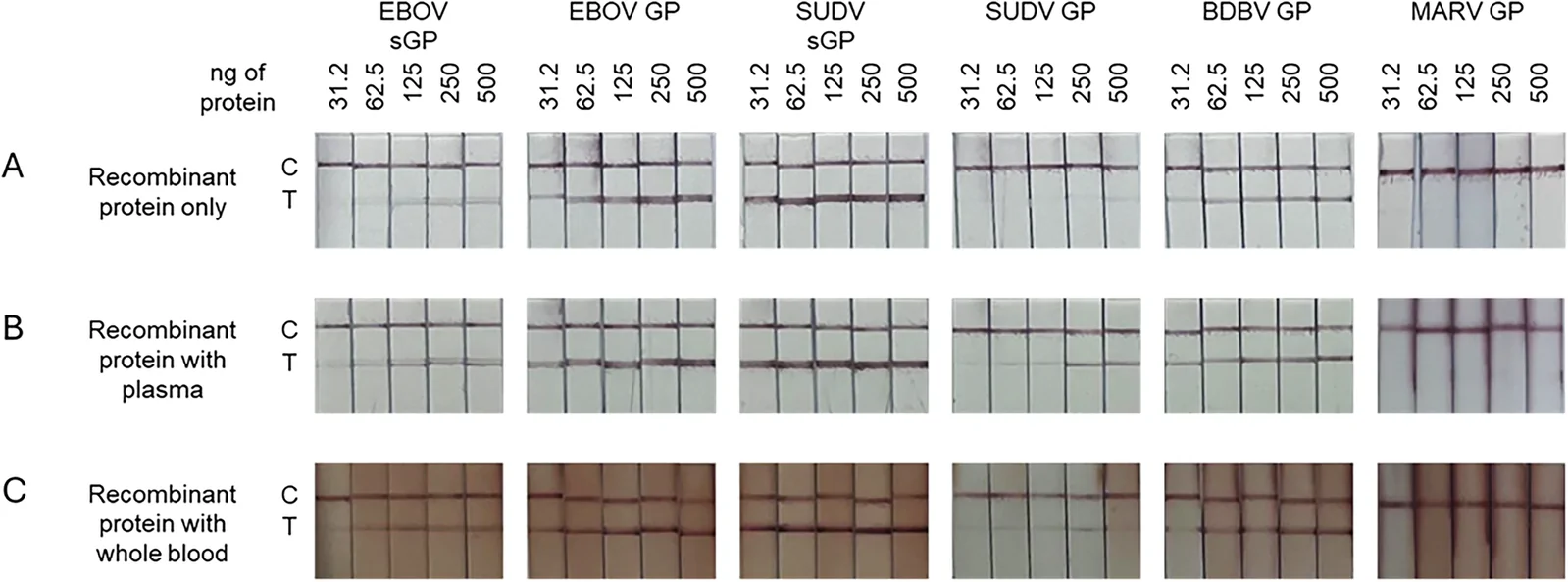
The pan-orthoebolavirus ILF-RDT detects low concentrations of recombinant glycoproteins. Recombinant glycoprotein (GP) or secreted glycoprotein (sGP) from EBOV, SUDV, BDBV, or MARV was serially diluted from 31.2 to 500 ng of protein in 100 μL sample running buffer and applied to the test alone (**A**) or spiked with 10 μL per test strip of plasma (**B**) or whole blood (**C**) from a human donor (donor 1). The control and test lines are indicated with C and T, respectively.

Prototype RDTs containing gold-conjugated 6H8 as detection antibody and 5A3 as capture antibody were assembled and tested first with recombinant orthoebolavirus GPs (Fig. 2). Each GP was two-fold serially diluted in sample running buffer, and a total of 500 to 31.2 ng of protein in a volume of 100 µL was applied to each RDT. Results were assessed within 5 to 15 min of sample application. Both EBOV and BDBV GP produced positive results down to 31.2 ng, while SUDV GP was more difficult to interpret, with positive results at 500 and 250 ng but equivocal results at lower amounts of recombinant protein (Fig. 2A). Orthoebolaviruses also produce a truncated and secreted version of the GP, known as sGP, that is expressed at high levels early during infection and is thought to act, at least in part, as an immune decoy^24^. Both EBOV and SUDV sGP were detected in this assay, with SUDV sGP producing particularly robust positive results at every dilution tested. Notably, the ILF-RDT was unable to detect GP from the more distantly related filovirus, Marburg virus (MARV), even when 500 ng of protein was applied to the assay, indicating that the test is specific for orthoebolaviruses.

We next sought to evaluate the performance of the prototype ILF-RDT in the presence of human plasma or whole blood, to ensure that these clinically relevant substrates would not interfere with test performance. Thus, each 100-µL sample of recombinant protein diluted in sample running buffer was spiked with human plasma (Fig. 2B) or whole blood (Fig. 2C) and re-evaluated. A volume of 10 µL of plasma or whole blood was chosen to reflect the actual volume that might be applied from a true patient sample^25^. Similar to the results with recombinant protein in running buffer only, the RDT detected EBOV GP, EBOV sGP, SUDV sGP, and BDBV GP at amounts as low as 62.5 or 31.2 ng. SUDV GP was clearly detected down to 250 ng, with equivocal results at lower amounts. Once again, MARV GP was not detected.

### The ILF-RDT detects authentic virus

**Fig. 3:**
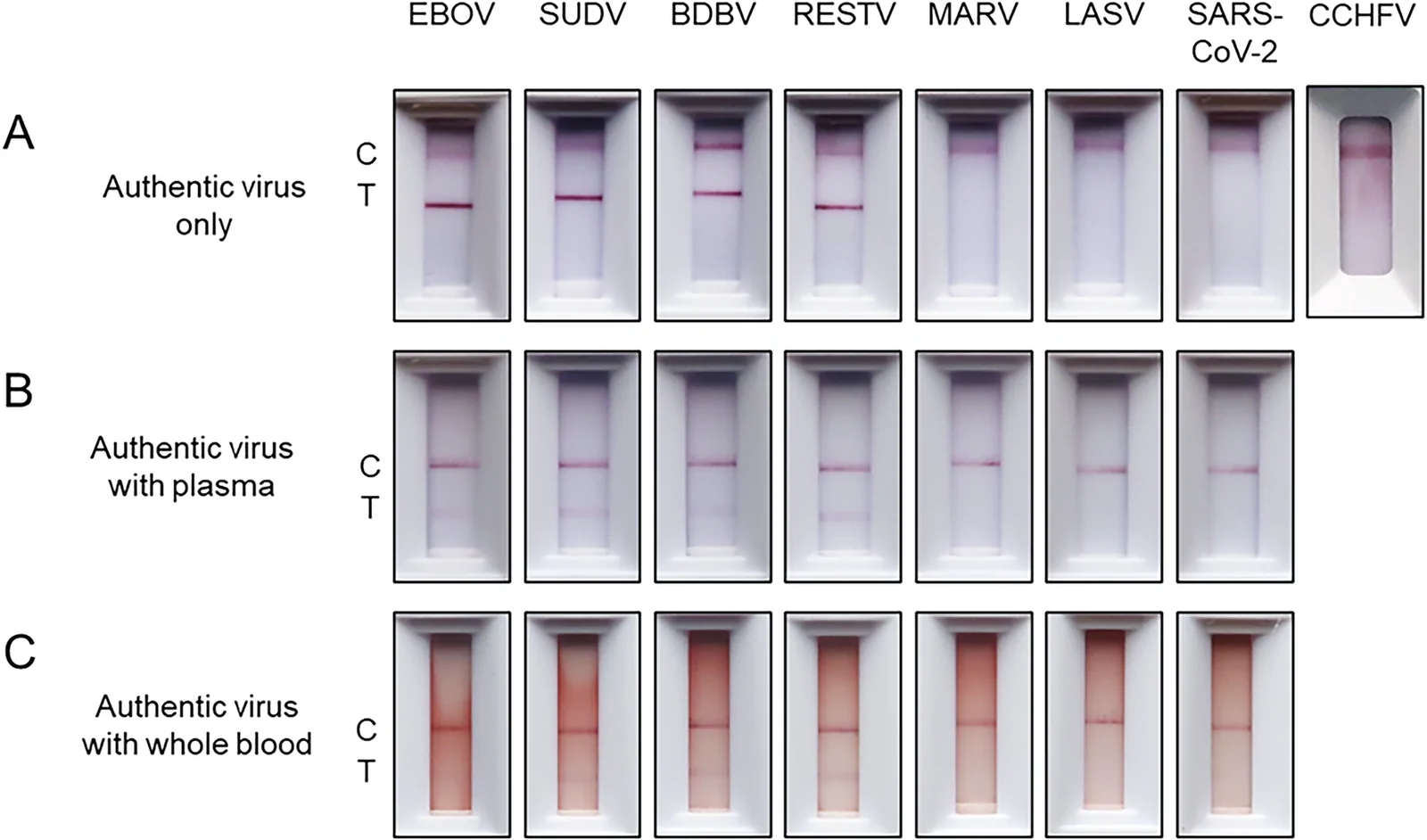
The pan-orthoebolavirus ILF-RDT detects authentic EBOV, SUDV, BDBV, and RESTV. EBOV (363,500 TCID_50_), SUDV (60,000 TCID_50_), BDBV (50,000 TCID_50_), RESTV (25,050 TCID_50_), MARV (158,000 TCID_50_), LASV (500,000 TCID_50_), SARS-CoV-2 (50,000 TCID_50_), or CCHFV (16,000 TCID_50_) in a volume of 50 μL was added to 50 μL of sample running buffer and applied to the test alone (**A**) or spiked with 5 μL of human plasma (**B**) or whole blood (**C**) from donor 1 per test strip. The control and test lines are indicated with C and T, respectively.

Given that the 6H8/5A3-based RDT could specifically detect recombinant orthoebolavirus GPs in a variety of substrates, we next sought to determine whether the test could detect authentic virus (Fig. 3). To that end, 50 µL of virus stock was applied directly to each RDT, followed by 50 µL of sample running buffer, and results were assessed within 30 min. EBOV (at a total of 363,500 TCID_50_), SUDV (60,000 TCID_50_), and BDBV (50,000 TCID_50_) all produced clear positive results (Fig. 3A), reflecting the results obtained with recombinant GPs (Fig. 2). The test also detected RESTV (25,050 TCID_50_), further demonstrating the breadth of this broadly cross-reactive RDT. MARV (158,000 TCID_50_) was not detected by the test and therefore served as a negative control, along with the unrelated viruses, Lassa virus (LASV; 500,000 TCID_50_), SARS-CoV-2 (50,000 TCID_50_), and Crimean-Congo Hemorrhagic Fever virus (CCHFV; 16,000 TCID_50_). When the 50-uL stocks of virus were spiked with 5 µL of human plasma (Fig. 3B) or whole blood (Fig. 3C) prior to application, all orthoebolaviruses produced a positive result, although the test lines were fainter than what was observed in sample buffer alone. A second set of evaluations, using 10 µL of whole blood from a different donor, revealed much stronger positive test results for all orthoebolaviruses assessed, including TAFV (Fig. S3). While aberrant background signal was observed in two of the four negative controls, this signal was markedly different (and less intense) than what was observed with the orthoebolaviruses. Whole blood alone, without virus, also produced a negative test result. Together, these results support the proof-of-principle efficacy of the diagnostic test, and they demonstrate that the presence of a clinically relevant substrate, such as whole blood, does not interfere with test performance.

**Fig. 4:**
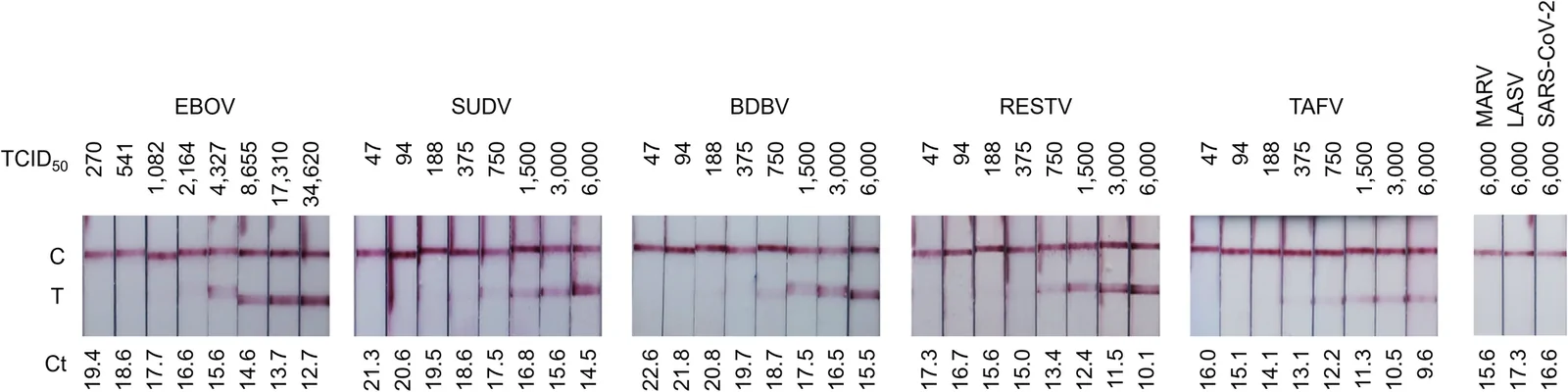
The pan-orthoebolavirus ILF-RDT detects low concentrations of authentic EBOV, SUDV, BDBV, and TAFV. The indicated viruses were serially diluted two-fold in sample running buffer from a starting concentration of approximately 6000 TCID_50_ or 34,620 TCID_50_ (EBOV only), and 100 μL of each dilution was applied to each test strip. The control and test lines are indicated with C and T, respectively. The cycle threshold (Ct) values from RT-qPCR assays targeting the L gene from RNA extracted from virus stocks are provided for comparison.

We assessed the sensitivity of the RDT by titrating down the concentration of each orthoebolavirus (Fig. 4). Virus was two-fold serially diluted in sample running buffer before 100 µL of each dilution was applied to each test. With the exception of EBOV, the maximum titer for each virus was ~6000 TCID_50_ and the minimum titer was 47 TCID_50_. Because the test proved to be less sensitive to EBOV, we evaluated higher titers of this virus, from 34,620 TCID_50_ to 270 TCID_50_. These assays revealed that relatively low amounts of virus could still be detected, with SUDV, BDBV, and RESTV detected down to 750 TCID_50_, which was equivalent to RT-qPCR Ct values of 17.5, 18.7, and 13.4. EBOV produced a positive result at a minimum of 4327 TCID_50_, equivalent to a Ct of 15.6. Notably, this assay also included TAFV, which was detectable down to 375 TCID_50_ (Ct of 13.1), further expanding the diagnostic breadth of this RDT. As expected, MARV, LASV, and SARS-CoV-2 were not detected by this assay.

The overall lower sensitivity of our ILF-RDT for EBOV led us to evaluate multiple different EBOV variants (Fig. S4). While EBOV variants Makona-C07 (used throughout the analyses in this paper), Kikwit, and Mayinga all produced positive results, Makona-C05 did not (Fig. S4A). GP sequence analysis revealed only a single amino acid difference between Makona-C07 and Makona-C05, namely a tryptophan to arginine mutation at position 291 (W291R) (Fig. S4B). Importantly, this mutation is rare: analysis of all 2985 available orthoebolavirus GP sequences in GenBank revealed that only 14 possessed the W291R substitution (Fig. S4C), with all 14 belonging exclusively to sequences of EBOV variant Makona-C05. A subsequent ELISA demonstrated that mAb 5A3, unlike 6H8, did not bind to the GP from Makona-C05 (Fig. S4D), suggesting that the W291R point mutation specifically obstructs binding to the assay’s capture mAb. Further analyses confirmed that the RDT detected both SUDV variant Boneface and Gulu (Fig. S5A) but did not detect MARV variants Angola or Musoke, nor did it detect Ravn virus (Fig. S5B).

### The ILF-RDT detects virus in experimentally inoculated animals

**Fig. 5:**
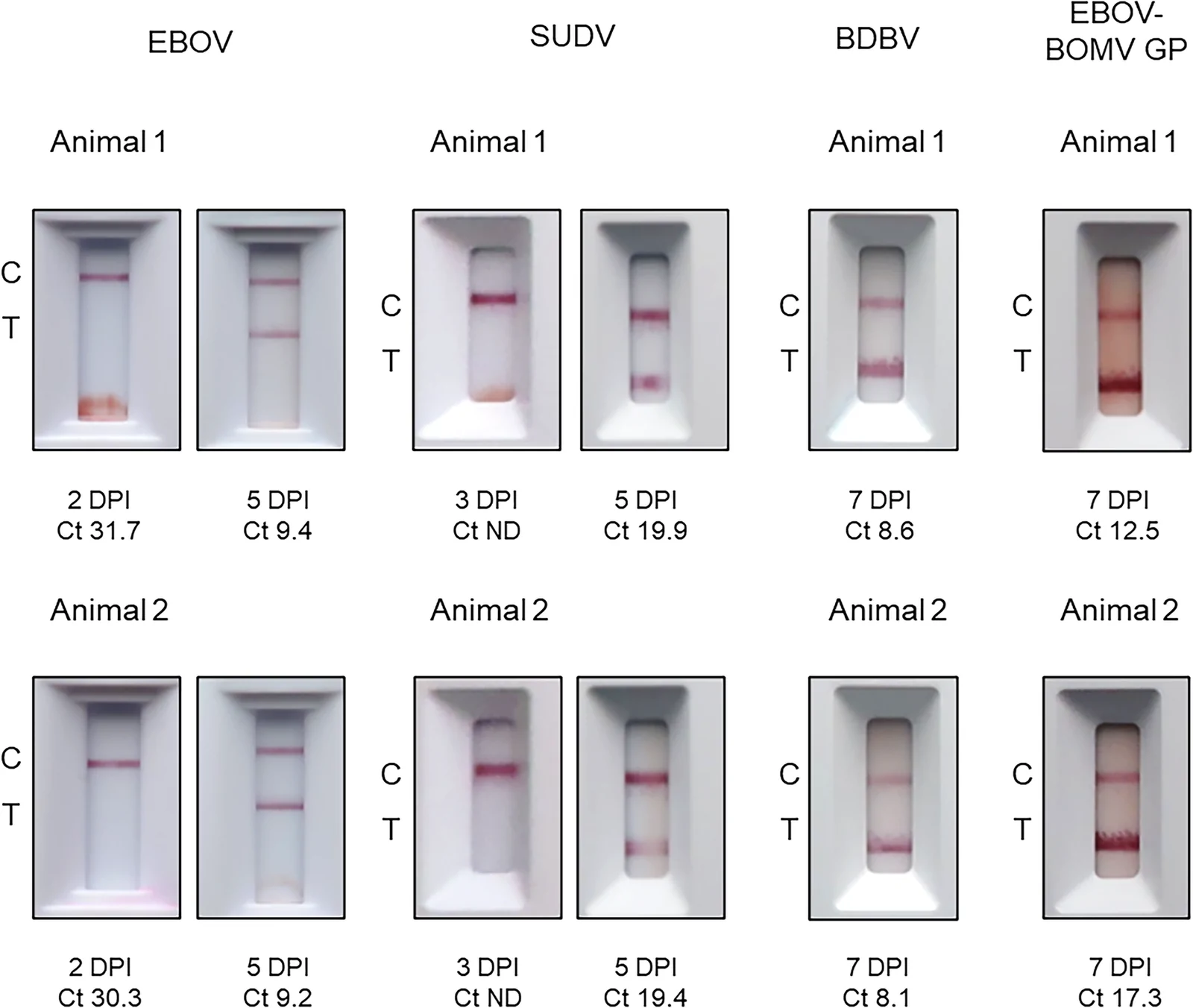
The pan-orthoebolavirus ILF-RDT detects virus in blood samples from experimentally inoculated ferrets. Whole blood samples from ferrets infected with EBOV, SUDV, BDBV, or EBOV-BOMV GP were tested at various days post-infection (DPI), as indicated. A total of 10 μL of whole blood was applied to the test strip and run with 100 μL of sample running buffer. The cycle threshold (Ct) values from RT-qPCR assays targeting the L gene from RNA extracted from whole blood samples are provided for comparison. ND not detected.

Given the ability of the ILF-RDT to detect EBOV, SUDV, BDBV, RESTV, and TAFV at relatively low levels, we next evaluated the assay using whole blood samples obtained from experimentally infected animals. Fresh blood samples were obtained from ferrets inoculated with either EBOV or SUDV, while banked (i.e., frozen) blood samples were obtained from ferrets inoculated with BDBV or a recombinant EBOV expressing the BOMV GP in place of the EBOV GP (EBOV-BOMV GP). In these studies, blood samples were taken from infected animals at pre-determined time points that were not necessarily chosen based on the intent to evaluate the ILF-RDT. A total of 10 µL of blood was applied directly to each RDT, followed by 100 µL of sample running buffer, and the tests were assessed within 15–30 min (Fig. 5). The two EBOV-infected animals showed clear positive results at 5 days post-infection (DPI), corresponding to a Ct value of ~9, when the animals were severely ill. In contrast, the tests were negative at 2 DPI, when the Ct values were greater than 30, and the animals were not exhibiting any clinical signs of infection. Similarly, the two SUDV-infected animals tested positive at 5 DPI (Ct ~19–20) when the animals showed severe signs of disease, but not at 3 DPI when the animals were relatively healthy (and viral RNA was not detected). Only a single timepoint—7 DPI—was assessed for samples from ferrets infected with BDBV or EBOV-BOMV GP, and both were positive, with Ct values around 8 for BDBV and between 12 and 17 for EBOV-BOMV GP, corresponding to severely ill animals.

**Fig. 6:**
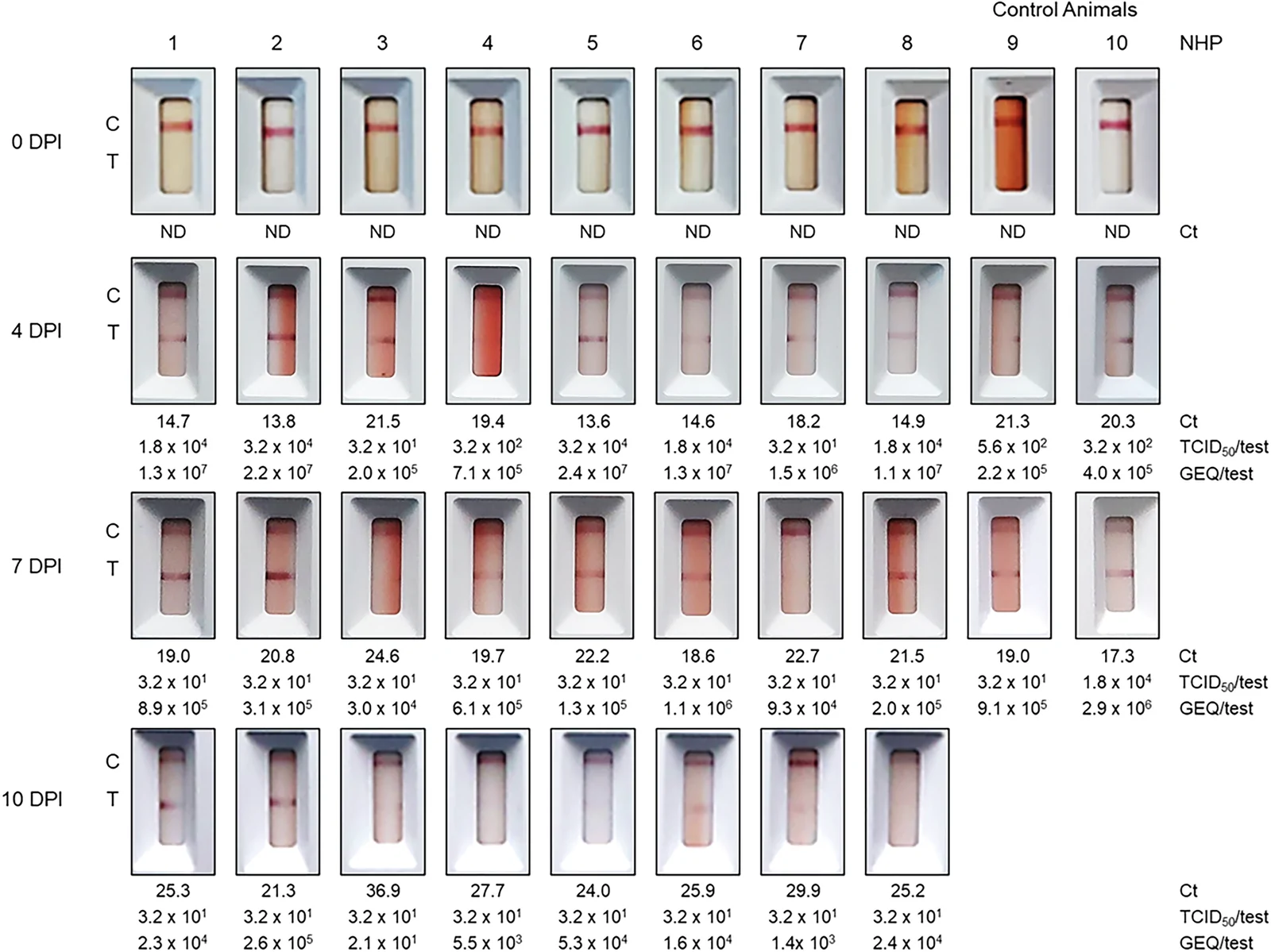
The pan-orthoebolavirus ILF-RDT detects virus in blood samples from experimentally inoculated nonhuman primates. Whole blood samples from nonhuman primates (NHPs) infected with EBOV and treated with an antibody cocktail were tested at various days post-infection (DPI), as indicated. A total of 10 μL whole blood was applied to the test strip and run with 100 μL of sample running buffer. The cycle threshold (Ct) values from RT-qPCR assays targeting the L gene from RNA extracted from whole blood samples are provided for comparison, along with the median tissue culture infectious dose (TCID_50_)/test and the genome equivalents (GEQ)/test. ND, not detected.

Finally, we assessed banked blood samples from ten cynomolgus macaques previously infected with EBOV and treated with a mAb cocktail (animals #1-8) or PBS (animals #9, #10) (Fig. 6). Once again, a total of 10 µL of blood was applied to each RDT and followed with 100 µL of sample running buffer. While all animals tested negative at 0 DPI, prior to virus inoculation, all tested positive at 4 DPI (with Ct values ~ 13–14), prior to the administration of the mAb treatment, albeit with varying levels of band intensity at the test line. Eight of 10 animals, including the untreated control animals, remained positive at 7 DPI (with Ct values ~ 19–22), with two other animals (#3 and #7) showing very faint bands at the test line. At 10 DPI, when the Ct values were much higher and the control animals had succumbed, two of the remaining eight mAb-treated animals (#1 and #2) still showed clear positive results, while two (#6 and #7) were faintly positive, and two (#3 and #5) were very faintly positive. Whether the treatment mAbs received by animals #1–8 might have negatively impacted the performance of our ILF-RDT (perhaps by occluding the binding sites for the mAbs present in the test) is currently unclear but cannot be ruled out. Notably, however, while the control animals and some of the mAb-treated animals were showing severe signs of EBOV disease at 7 DPI, they were only just beginning to show mild signs of illness at 4 DPI, such as decreased activity and inappetence. The fact that the ILF-RDT was able to detect EBOV as early as 4 DPI suggests that the RDT may be able to detect virus prior to the manifestation of severe disease.

## Discussion

RDTs have an important role to play in the diagnosis of EBOD and the management of orthoebolavirus outbreaks. While these tests cannot replace more sensitive molecular diagnostic assays, such as RT-qPCR, they can greatly augment frontline diagnostic capacity. With specific and rapid point-of-care testing provided by an RDT, suspected cases could be identified earlier and triaged immediately, even in remote or resource-poor locations with limited access to advanced diagnostic capabilities. Indeed, the widespread use of inexpensive and simple to operate RDTs on the frontlines of an orthoebolavirus outbreak, or within orthoebolavirus endemic zones, may ultimately help to stem the spread of disease and ensure that infected individuals receive safe and appropriate care as soon as possible. Such a diagnostic assay may have been especially useful in the early stages of the ongoing BDBV outbreak in the DRC, where the remoteness of the initial cases along with the lack of a BDBV-specific diagnostic tests likely delayed recognition of the virus’s re-emergence.

Our ILF-based RDT is unique in its ability to rapidly and specifically detect all known orthoebolavirus GPs, as well as EBOV and SUDV sGPs, making it a potentially valuable new diagnostic tool. Not only can this ILF-RDT detect the human-pathogenic orthoebolaviruses—EBOV, SUDV, BDBV, and TAFV—in a variety of substrates, including whole blood, but it can also detect RESTV and a recombinant EBOV expressing BOMV GP. While the utility of detecting RESTV and BOMV—which are not known to cause disease in humans—may be limited in the context of public health, the test could prove useful for secondary purposes, including screening animals known to harbor or be infected by filoviruses, such as bats and pigs. We speculate that the ability to detect sGP, which is produced in high quantities relatively early during orthoebolavirus infection^24^, likely contributes to the assay’s sensitivity and may allow the test to detect positive cases earlier during the disease course. Additionally, the fact that this ILF-RDT detects all known orthoebolavirus GPs suggests that it may also detect yet-to-be discovered orthoebolaviruses, which could prove useful as the filovirus family continues to expand and outbreaks continue to occur.

Over the course of developing this pan-orthoebolavirus ILF-RDT, we performed extensive and iterative analyses to refine each step of the assay, and we encountered four distinct challenges. The first challenge involved the order in which the mAbs bound to the antigen, and this required testing each antibody as both a detection mAb and a capture mAb. We noted that using 6H8 as the detection mAb, which binds to GP first, followed by 5A3 as the capture mAb, offered significantly more sensitivity than the reverse pairing, likely because binding of 5A3 occludes subsequent binding of 6H8. Future work will be focused on determining the epitopes of each antibody in order to completely understand how the mAbs interact with GP. The second challenge involved the frequency with which the test produced false positive results, especially when using uninfected human and NHP blood. To address this issue, we added urea to the sample buffer for all tests. The addition of urea resolved the false positives—likely by reducing non-specific binding and the formation of aggregates—but did not impact the ability of the assay to detect recombinant GP or authentic virus. Further modifications to the sample buffer and the constituent parts of the ILF test (e.g., the nitrocellulose membrane, conjugate release pads, sample application and absorption pads, etc.) may help reduce the reliance on urea. In the future, it will also be important to evaluate blood samples that do not contain EDTA, an anticoagulant and chelating agent, since it may interfere with some ILF assays^26,27^. The third challenge we encountered related to the differences in assay performance that were observed between different lots of the test strips. Since we engineered these strips in small, bespoke batches, slight differences in reagent composition or assembly material might have affected test performance. Large batch preparation with dedicated quality control protocols may help to reduce this variability in the future. Finally, the fourth challenge involved the use of recombinant GPs for initial screening and preliminary analysis of the ILF-RDT assays. Recombinant antigens can vary in quality and may not fully represent the native state of the glycoprotein, which could impact the perceived sensitivity of the test. This may explain, at least in part, why the results with recombinant SUDV GP (Fig. 2) appeared much less sensitive than the results using authentic SUDV (Figs. 3–5). Thus, confirming test performance using authentic virus, including clinically relevant samples from experimentally infected animals, was critical to assay development. In fact, the sensitivity of the ILF-RDT for EBOV increased when using blood samples from infected NHPs (Fig. 6) compared to stock virus (Fig. 4), with positive test results achieved in the former set of samples at higher Ct values than the latter. We suspect that this discrepancy may reflect increased levels of sGP or shed GP in blood samples from infected animals compared to virus stocks grown in cell culture, but this remains the subject of further investigation.

An intriguing but important limitation of this ILF-RDT is that it did not detect EBOV variant Makona-C05, which we presume is the result of a tryptophan to arginine substitution in GP at position 291 (W291R) that prevented binding of 5A3. Despite occurring within the glycan cap of GP, which has relatively low conservation between orthoebolavirus species, the region around and including residue 291 is highly conserved across all orthoebolaviruses (Fig. S4B), with the W291R substitution found only in the EBOV Makona-C05 variant and appearing in only 0.5% of all publicly available orthoebolavirus sequences. Given the highly conserved nature of W291, it is unlikely that the inability of our assay to detect R291 would have a significant impact on the utility of this test, which is still capable of detecting the full diversity of different viruses within the *Orthoebolavirus* genus. However, this example does illustrate the degree to which test sensitivity relies on antibody specificity, and future work will focus on adapting this test to ensure recognition of EBOV Makona-C05, either by incorporating an additional capture mAb or by modifying 5A3.

A number of different ILF-based RDTs have been developed for orthoebolaviruses, each with distinct properties that differ from our ILF-RDT^13,21,28,29^. The most well characterized and widely recognized of these RDTs are ReEBOV^30–32^, OraQuick™ Ebola^33,34^, QuickNavi-Ebola^25,35^, and EBOLA Ag K-SeT^36^ (Table S1). The ReEBOV and OraQuick™ Ebola RDTs both specifically detect the VP40 matrix proteins of EBOV, SUDV, and BDBV in whole blood, serum, and plasma (ReEBOV) or whole blood and oral fluids (OraQuick™ Ebola)^30–34^. Similarly, the EBOLA Ag K-SeT detects EBOV VP40 in whole blood, serum, and plasma^36^. The QuickNavi-Ebola RDT, in contrast, detects the nucleoprotein (NP) of EBOV, SUDV, and TAFV in whole blood and serum^25^. Thus, our test differs from these established tests in that it detects the viral GP of all known orthoebolaviruses. We chose to target GP for our assay to capitalize on three distinct advantages: (1) GP is present on the surface of the virions and may therefore be more accessible to the assay’s antibodies, thus increasing sensitivity; (2) sGP is produced in abundance during orthoebolavirus infection, thus making it an attractive diagnostic target that might also increase assay sensitivity; and (3) despite antigenic differences among different GPs, these proteins also show a high degree of sequence conservation in some areas, which is conducive for the development of pan-reactive assays. Indeed, the success of our ILF-RDT provides the proof-of-principle for this approach.

Although a range of reactivity to a limited number of different orthoebolaviruses has been demonstrated for most of the established RDTs, large scale validation has mainly been performed for EBOV. Direct comparisons of test sensitivity (i.e., the ability to identify true positives) and test specificity (i.e., the ability to identify true negatives) across different RDT platforms is difficult owing to the different ways in which validations have been performed; however, a recent meta-review offers substantial insight^28^. The pooled specificity of the ReEBOV, OraQuick^TM^ Ebola, QuickNavi-Ebola, and EBOLA Ag K-Set tests was high, ranging from ~90 to ~99%, with the OraQuick™ Ebola RDT offering the best results. Pooled sensitivity, on the other hand, was generally lower and more variable, ranging from ~54 to ~90%, with the ReEBOV RDT achieving the highest sensitivity. Whether our ILF-RDT is capable of matching or surpassing the specificity and sensitivity of these other RDTs is currently unknown. Notably, while we have clearly established proof-of-principle for our novel ILF-RDT, we have yet to evaluate its performance under “real world” conditions, which represents another important limitation of this study. Indeed, further optimization of this test will require not only the evaluation of human clinical samples but also evaluation in the field, outside of ideal laboratory conditions. Future work will be focussed on this type of validation, as and when we gain access to these rare and precious clinical samples.

In the absence of specificity/sensitivity validation, we can approximate test performance based on an analysis of assay limits of detection (LOD). Although we did not conduct a systematic evaluation of our assay’s LOD, we estimate from our data (Fig. 4) that these values range from 375 TCID_50_ for TAFV to 750 TCID_50_ for SUDV, BDBV, and RESTV and 2164 TCID_50_ for EBOV. Because differences in virus quantification assays among different labs can result in dramatically different titers, care must be taken when comparing LODs across different RDTs. However, at least superficially, the estimated LODs from our ILF-RDT align with those of the other RDTs, which range from 30 to 300 FFU per test for QuickNavi-Ebola to 30,000 PFU for ReEBOV and 32,800 TCID_50_ for OraQuick™ Ebola^25,30,32–34^ (Table S1). Future work will focus on calculating robust LOD levels for our ILF-RDT based on both infectious virus titers and recombinant protein concentrations; however, given our preliminary results, we suspect that our test is at least as sensitive as existing tests, with the added and unique advantage of being able to detect all known orthoebolavirus GPs.

According to a target product profile developed by the WHO during the 2013–2016 EBOV outbreak in West Africa, RDTs should target patients presenting with a fever at the point-of-care, provide a clinical sensitivity of at least 98% and a specificity of greater than 99% using whole blood, and deliver results in less than 30 min^21^. Ideally, the assays should not require extensive training, nor should they depend on a consistent power source. While no existing RDT has yet achieved these standards—specifically with respect to test sensitivity and specificity—it is clear that an RDT could serve as a valuable tool for quickly identifying positive cases and thus limiting the spread of virus. Although more work is required to optimize and validate our prototype pan-orthoebolavirus ILF-RDT, such a broadly cross-reactive test could prove especially useful at the forefront of outbreak management and throughout areas where multiple different orthoebolaviruses regularly circulate and emerge.

## Methods

### Animal ethics and biosafety statement

Animal work was conducted at the Canadian Science Centre for Human and Animal Health (CSCHAH), National Microbiology Laboratory, Public Health Agency of Canada in Winnipeg, Manitoba, following protocols (H-16-026, H-20-004, H-20-008 H-23-009) approved by the Institutional Animal Care Committee in accordance with guidelines from the Canadian Council on Animal Care. All work with viruses was conducted in the containment level 4 laboratory at the CSCHAH in accordance with standard operating protocols.

### Antibodies and recombinant proteins

All orthoebolavirus-specific mAbs were generated and characterized as previously described^23^. An anti-mouse IgG antibody (Sigma, Product # M8642) was used for the control line of the RDT. Recombinant proteins used in this study for determining reactivity of antibodies by ELISA, binding kinetics by BLI, and development of the ILF assay are listed in Table S2.

### Viruses

The following orthoebolaviruses were used: Ebola virus variants Makona-C07 (EBOV; Ebola virus/H.sapiens-wt/GIN/2014/Makona-Gueckedou-C07; GenBank accession no. KJ660347.2), Makona-C05 (Ebola virus/H.sapiens-wt/GIN/2014/Makona-Gueckedou-C05; GenBank accession no. KJ660348.2), Kikwit (Ebola virus/H.sapiens-tc/COD/1995/Kikwit-9510621; GenBank accession no. AY354458.1), and Mayinga (Ebola virus/H.sapiens-tc/COD/1976/Yambuku-Mayinga; GenBank accession no. NC_002549.1); Sudan virus variants Gulu (SUDV; Sudan virus/H.sapiens-tc/UGA/2000/Gulu-808892; GenBank accession no. NC_006432) and Boneface (GenBank accession no. FJ968794.1); Bundibugyo virus (BDBV; Bundibugyo virus/H.sapiens-tc/UGA/2007/Butalya-811250; GenBank accession no. NC_014373); Taï Forest virus (TAFV; Tai Forest virus/H.sapiens-tc/CIV/1994/Pauleoula-CI; GenBank accession no. NC_014372.1); and Reston virus (RESTV; Reston virus/M.fascicularis-tc/USA/1989/Philippines89-Pennsylvania; GenBank accession no. NC_004161.1). A recombinant EBOV/Makona-C07 expressing Bombali virus GP instead of the endogenous GP (EBOV-BOMV GP) was recovered from cDNA, essentially as previously described^37^. Additional viruses used as negative controls included: Marburg virus variants Angola-368 (identical to GenBank accession no. KY047763), Angola-325 (Marburg virus/H.sapiens-tc/ANG/2004/Angola325; GenBank accession no. PX278685), and Musoke (Marburg virus/H. sapiens-tc/KEN/Mt. Elgon-Musoke F3; GenBank accession no. PX278686); Ravn virus (RAVV; Ravn virus/H. sapiens-tc/KEN/1987/Ravn-CDC811103; GenBank accession no. KU179482.1); Lassa virus (LASV; Lassa virus/H.sapiens-wt/NGA/2018/NML_46; GenBank accession nos. MZ169792, MZ169793); SARS-CoV-2 (hCoV-19/Canada/UN-295389-p1/2022; GISAID EPI_ISL_13227179; BA.2); and Crimean-Congo Hemorrhagic Fever virus (CCHFV) strain Kosovo Hoti. Infectious virus titers were calculated as median tissue culture infectious dose (TCID_50_), as described previously^38^.

### Blood samples

Human EDTA whole blood and plasma were purchased from StemCell Technologies, which collected the samples using consent forms and protocols approved by either an Institutional Review Board, the Food and Drug Administration, the U.S. Department of Health and Human Services, and/or an equivalent regulatory authority. “Donor 1” (Figs. 2 and 3) was a 27-year-old white, female donor with a blood type of B+ (Catalogue # 200-0151, Lot # 1000166095, Donor # CE0010253), and “Donor 2” (Fig. S3) was a 25-year-old white, female donor with blood type of A+ (Catalogue # 200-0150, Lot # 1000273263, Donor # RG3652). EDTA whole blood samples were obtained from groups of two ferrets each inoculated with 1000 TCID_50_ EBOV/Makona-C07, SUDV/Gulu, BDBV, or EBOV-BOMV-GP. Samples from EBOV- and SUDV-infected ferrets were fresh (not frozen), while samples from BDBV- and EBOV-BOMV-GP-infected ferrets had been frozen at -80 °C prior to use. EDTA whole blood samples, which had been previously frozen at −80 °C, were also obtained from ten cynomolgus macaques inoculated with 1000 PFU EBOV/Makona-C07.

### Antibody binding competition analysis

Antibody binding competition was assessed using biolayer interferometry (BLI) performed on the Octet RED 96 (ForteBio, Inc) instrument with Octet SA Biosensors (Sartorius, Product # 18-5019). All samples were diluted in 1× kinetics buffer (Sartorius, Product # 18-1105). mAbs were first biotinylated with the BiotinTag Micro Biotinylation Kit (Sigma-Aldrich, Product # BTAG) according to the manufacturer’s instructions, and quantified using the Qubit Protein Assay Kit (ThermoFisher Scientific, Product # Q33212) on a Qubit 4 Fluorometer (ThermoFisher Scientific). For binding competition, Octet SA Biosensors were equilibrated in 1× kinetics buffer and loaded with biotinylated competitor mAbs at 2.5 μg/mL for 120 s, followed by a second equilibration for 60 s. EBOV, SUDV, or BDBV GP was captured onto sensors at 500 nM for 360 s. After a brief equilibration for 60 sec, binding of probing mAbs was assessed for 180 s followed by dissociation for 120 s. A reference sensor loaded without mAb and subjected to association without GP was included as background. Data were analyzed to generate binding competition matrices using Octet Data Analysis software version 13.0.2.46. The data portion for probing mAb binding and dissociation were plotted using GraphPad Prism version 10.4.2.

### Preparation and assembly of ILF assay strips

To prepare detection antibodies, mAbs were desalted and conjugated to 80 nm carboxyl gold nanospheres using the BioReady Covalent Gold Conjugation Kit for Lateral Flow, per the manufacturer’s instructions (nanoComposix, Product # AUZR80), yielding 20 μg/mL of conjugated antibody. Capture mAbs were diluted to 1 mg/mL in phosphate buffered saline (PBS), and the control antibody, goat anti-mouse IgG (Sigma, Product # M8642), was reconstituted to 1 mg/mL in 0.135 M NaCl.

Capture mAbs and control antibody were sprayed onto a nitrocellulose membrane pre-applied to a polystyrene backing card (Cytiva, Whatman FF120HP, Product # 10547021) to form the test and control lines, respectively, using a Linomat 5 instrument (CAMAG) fitted with 100 μL syringes. The antibodies were applied at 150 nL/second across a line ~1–2 mm in width at a density of 1 µg/cm. The prepared nitrocellulose membranes were then cured at 37 °C for 2 h and stored in a desiccator at room temperature with relative humidity below 20%. The conjugate release pad (Ahlstrom, Grade 6614) was pre-treated by immersion with a solution of PBS containing 10% sucrose, 0.5% bovine serum albumin, and 0.1% Triton X-100, cured and stored as described above until further use. The gold-conjugated detection mAbs were applied to the pre-treated conjugate release pad using a Linomat 5 instrument fitted with a 100 μL syringe at a speed of 1 μL/second across an area ~6–8 mm in width to a density of 0.2 µg/cm. The prepared conjugate release pad was cured and stored as described above.

To assemble the test strips, an absorption pad (Cytiva, Whatman GF/DVA) was applied to the backing card, overlapping the distal end of the prepared nitrocellulose membrane by ~2 mm. The prepared conjugate release pad was applied at the opposite end, overlapping the nitrocellulose membrane by ~2 mm, after which the sample application pad (Ahlstrom, Grade 1668 HV + ), which also served as the blood separation pad, was applied, overlapping the conjugate release pad by ~ 2 mm. All components were laminated together using a Matrix 2210 Universal Laminator Module (Kinematic Automation Inc). Laminated cards were cut into 4-mm wide strips using a Matrix 2360 Programmable Shear (Kinematic Automation Inc). Individual test strips were loaded into cassettes (Drummond Scientific, Product # DRU-6-000-0250) and stored in a desiccator, as above. For longer term storage, test strips were vacuum sealed with desiccant and stored at 4 °C. All samples were run in sample running buffer, composed of 40 mM Tris, 300 mM NaCl, 500 mM urea, 0.5% BSA, 0.5% Tween 80 and adjusted to pH 8. For blood samples from NHPs, the urea concentration was adjusted to 1 M.

### RNA extraction and determination of viral RNA load

RNA was extracted from neat and diluted viral stocks and whole blood of infected animals using the QIAamp Viral RNA Mini Kit (Qiagen, Product # 52906) per the manufacturer’s instructions and subsequently stored at -80 °C. RT-qPCR was performed to determine the viral RNA load using TaqPath™ 1-Step Multiplex Master Mix (Applied Biosystems, Product # A28523) on either a QuantStudio™ 3 Real-time PCR System (Applied Biosystems) or a LightCycler® 96 Instrument (Roche). Data was analyzed using either the QuantStudio™ Design & Analysis Software version 1.5.3 (Applied Biosystems) or LightCycler® 96 Software version 1.1 (Roche) depending on the instrument used. A total reaction volume of 25 µL was prepared consisting of 6.25 µL of 4X master mix, 0.5 µL of 20 µM forward primer, 0.5 µL of 20 µM reverse primer, 0.5 µL of 10 µM double-quenched probe (IDT Inc., Canada), 12.75 µL of nuclease-free water, and 5 µL of RNA. Primer and probe sequences targeting the polymerase (L) gene of EBOV, SUDV, BDBV, TAFV, and MARV were taken from ref. ^14^, and for LASV, from ref. ^39^. For RESTV, RES_F (5ʹ-GGAAGCGAGAGTCAACCTTAG-3ʹ), RES_R (5ʹ-CGGGCTGTATTGGTCGTTAT-3ʹ), and RES_P (5ʹ-[FAM]-TCAGTGAAG[ZEN]TCCTGCAAATGACACCA-[3IABkFQ]-3ʹ) (IDT Inc., Canada) were used to target the L gene. Cycling was performed in standard mode, with parameters as follows: 53 °C for 10 min, 95 °C for 2 min, followed by 45 cycles of 95 °C for 3 s, and 60 °C for 30 s at which time fluorescent signal was detected. For SARS-CoV-2, the envelope (E) gene was targeted and the assay performed using SuperScript™ III Platinum™ One-Step qRT-PCR Kit (Invitrogen, Product # 11732088) as previously described^40^. Thresholds were determined automatically by the instrument’s software based on a positive control and was applied to each sample to determine the cycle threshold (Ct) value. Genome equivalents (GEQ) were calculated using a standard curve generated from a plasmid encoding EBOV L.

### Protein sequence analysis

All orthoebolavirus GP protein sequences greater than 400 amino acids were downloaded from GenBank on 07 May 2025 and aligned using Muscle 5.1 in Geneious Prime 2025.0.3 (https://www.geneious.com) to determine the frequency of tryptophan and arginine residues at position 291. The sequence alignment of this region for all tested orthoebolaviruses was visualized using the package ggmsa version 1.12.0^41^ using R version 4.4.1^42^.

## Supplementary information

Supplementary information

## Data Availability

All data generated or analyzed during this study are included in this published article and its supplemental information files. The raw image files for Figures 2-6, S3-5 can be found in the supplementary information (Fig. S6-S13).
