# Supplementary material for: Development of an immunochromatographic lateral flow assay for the rapid detection of all known orthoebolavirus glycoproteins
Source: Npj Viruses. 2026 Jun 24;4:48. doi: 10.1038/s44298-026-00209-1 (PMC13658771; doi:10.1038/s44298-026-00209-1)
Supplement: Supplementary file 1 [file 44298_2026_209_MOESM1_ESM.docx]

***Schulz et al. A pan-orthoebolavirus rapid diagnostic test***

**SUPPLEMENTARY INFORMATION**

**Figure S1. Schematic of an immunochromatographic lateral flow (ILF) rapid diagnostic test (RDT).** (A) Sample is applied to the sample application pad and wetted with running buffer. Capillary action driven by the absorption pad pulls the sample across the test strip. Sample contacts the detection antibody (conjugated to a signal-producing moiety, such as gold nanoparticles) in the conjugate release pad. Antigen in the sample is bound by the detection antibody prior to entering the nitrocellulose membrane, where the immune complex encounters the capture antibody immobilized at the test line. Binding of the antigen-detection antibody immune complex by the capture antibody at the test line results in the concentration of the signal-producing moiety, which produces a line that can be visualized by eye. Excess and unbound detection antibody continues to flow along the nitrocellulose membrane until it binds to the immobilized immunoglobulin-specific antibody at the control line, forming a second visible band and confirming correct flow of the assay. A positive result is indicated by two visible lines, while a negative result is indicated by a control line only. (B) Location of each of these components in the ILF-RDT and (C) how they bind and interact when the assay is being performed. Antigen is represented as red triangles, the detection mAb 6H8 conjugated to gold nanoparticles is shown as black antibodies attached to yellow spheres, the capture mAb 5A3 immobilized on the test line is shown as orange antibodies, and the goat anti-mouse immobilized on the control line is shown as grey antibodies. This figure was created using Microsoft^®^ PowerPoint^®^ version 2511**.** The icons used in panels B and C were created in [BioRender.com](https://BioRender.com) (Created in BioRender. Wight, J. (2026) <https://BioRender.com/dd13m19>; agreement number EP29TI5ZJ5 and EI29TI5ZQP).

**Figure S2.** **Bio-layer interferometry (BLI) using the Sartorius Octet instrument confirmed the selection of 5A3 as capture mAb and 6H8 as detection mAb for the pan-orthoebolavirus ILF test.** Streptavidin (SA) sensors loaded with biotinylated mAbs (representing gold-conjugated detection mAbs) were exposed to 500 nM recombinant EBOV, SUDV, or BDBV glycoprotein (GP), followed by exposure to a second, unconjugated mAb (representing the capture mAb). The association and dissociation of the second mAb was quantified. Values in this figure are the maximum binding signal of the second mAb at the end of the association step, with higher values indicating that binding of the first antibody does not restrict binding of the second.

**Figure S3. The pan-orthoebolavirus ILF-RDT detects authentic EBOV, SUDV, BDBV, TAFV, and RESTV in a different donor.** EBOV (363,500 TCID_50_), SUDV (60,000 TCID_50_), BDBV (50,000 TCID_50_), TAFV (6,300 TCID_50_) RESTV (25,050 TCID_50_), MARV (158,000 TCID_50_), LASV (500,000 TCID_50_), SARS-CoV-2 (50,000 TCID_50_), or CCHFV (16,000 TCID_50_) in a volume of 50 μL was added to 50 μL of sample running buffer containing 2000 mM urea and applied to the test alone (A) or spiked with 10 μL of human plasma (B) or whole blood (C) from donor 2 per test strip. The control and test lines are indicated with C and T, respectively.

**Figure S4. The pan-orthoebolavirus ILF-RDT does not detect EBOV variant Makona**-**C05.** (A) EBOV variants Mayinga (150,000 TCID_50_), Kikwit (64,700 TCID_50_), Makona-C07 (72,700 TCID_50_), and Makona-C05 (280,000 TCID_50_) were diluted 1:1 with sample running buffer, and 100 μL were applied to the ILF test. (B) A segment of the glycoprotein amino acid sequence alignment is shown for a representative subset of orthoebolaviruses, highlighting the unique polymorphism W291R in Makona-C05 that presumably prevents detection by the ILF test. (C) This W291R polymorphism is only present in EBOV variant Makona-C05, which accounts for a total of 14 (0.5%) orthoebolavirus sequences deposited in the NCBI data base. (D) To resolve which antibody cannot bind the Makona-C05 glycoprotein (GP), 96-well half-well ELISA plates were coated with 1x10^5^ TCID_50_ of gamma-irradiated Ebola virus Makona-C07 or Makona-C05. Following our standard ELISA protocol (ref. 22), the primary antibodies 5A3 and 6H8 were applied in duplicate at 1 μg/mL and secondary HRP-conjugated goat anti-mouse IgG (H) Ab (Southern Biotech Product # 1030-05) was applied at 1:2,000. The plates were read at an optical density (OD) of 650 nm, and values were corrected to the baseline reading.

**Figure S5. The pan-orthoebolavirus ILF-RDT detects SUDV variants but not MARV variants or RAVV.** For each test strip, 50 μL of stock virus was applied with 50 μL of sample running buffer. SUDV variants Boneface (6,000 TCID_50_) and Gulu (6,000 TCID_50_) were positive (A), while MARV variants Angola (158,000 TCID_50_) and Musoke (28,100 TCID_50_) were negative, as was Ravn virus (RAVV; 232,200 TCID_50_), were negative (B).

**Figure S6. Raw images corresponding to Figure 2.**

**Figure S7. Raw images corresponding to Figure 3.**

**Figure S8. Raw images corresponding to Figure 4.** Note that images were flipped left-to-right for presentation in Figure 4.

**Figure S9. Raw images corresponding to Figure 5.**

**Figure S10. Raw images corresponding to Figure 6.**

**Figure S11. Raw images corresponding to Figure S3.**

**Figure S12. Raw images corresponding to Figure S4.**

**Figure S13. Raw images corresponding to Figure S5.**

| **Table S1: Comparison of other orthoebolavirus RDTs** | | | |
| --- | --- | --- | --- |
| **RDT** | **Target** | **Viruses Detected** | **Limits of Detection** |
|  |  |  | **Virus Titers** |
| ReEBOV ^30-32^ | VP40 | EBOV | 3 x 10^4^ PFU/test |
|  |  | SUDV | 1.1 x 10^3^ PFU/test |
|  |  | BDBV | 9.5 x 10^1^ PFU/test |
|  |  | RESTV | Not reactive |
| OraQuick™ Ebola ^33,34^ | VP40 | EBOV | 3.28 x 10^4^ TCID_50_/test |
|  |  | SUDV | 5.6 x 10^4^ – 1.19 x 10^6^ PFU/test |
|  |  | BDBV | 3.98 x 10^4^ – 1.37 x 10^6^ PFU/test |
|  |  | RESTV | Not reactive |
|  |  | TAFV | Not reactive |
| QuickNavi-Ebola ^25,35^ | NP | EBOV | 3 x 10^1^ – 3 x 10^2^ FFU/test |
|  |  | SUDV | Not reactive |
|  |  | BDBV | 3 x 10^1^ FFU/test |
|  |  | RESTV | Not reactive |
|  |  | TAFV | 3 x 10^2^ FFU/test |
| Coris^®^ EBLOA Ag K-SeT ^36^ | VP40 | EBOV | Not reported |
| This study’s ILF-RDT | GP | EBOV | 2.16 x 10^3^ TCID_50_/test |
|  |  | SUDV | 7.5 x 10^2^ TCID_50_/test |
|  |  | BDBV | 7.5 x 10^2^ TCID_50_/test |
|  |  | TAFV | 3.75 x 10^2^ TCID_50_/test |
|  |  | RESTV | 7.5 x 10^2^ TCID_50_/test |
|  |  | BOMV | Not determined |
|  | | | |

| **Table S2: List of recombinant glycoproteins (GPs)** | | | |
| --- | --- | --- | --- |
| **Name** | **Vendor** | **Product #** | **Use** |
| EBOV GP∆TM | IBT | 0501-001 | ILF assay |
| SUDV GP∆TM | IBT | 0502-001 | ILF assay |
| BDBV GP∆TM | IBT | 0505-015 | ILF assay |
| EBOV sGP | IBT | 0565-001 | ILF assay |
| SUDV sGP | IBT | 0570-001 | ILF assay |
| MARV GP∆TM | IBT | 0503-001 | ILF assay |
| EBOV GP∆TM | IBT | 0501-015 | BLI |
| SUDV GP∆TM | IBT | 0502-025 | BLI |
| BDBV GP∆TM | IBT | 0505-015 | BLI |
